# Altered extracellular matrix remodeling accompanies decreased lncRNA HOTAIR expression in Takayasu arteritis

**DOI:** 10.3389/fimmu.2026.1800351

**Published:** 2026-04-16

**Authors:** Fernanda Espinosa-Bautista, María G. Soberanes-García, Diana Castillo-Martínez, Rashidi Springall, Adrián Hernández-Díazcouder, Luis M. Amezcua-Guerra

**Affiliations:** 1 Immunology Department, Instituto Nacional de Cardiología Ignacio Chávez, Mexico City, Mexico; 2 Dermatology Clinic, Hospital General de Zona No. 32, Instituto Mexicano del Seguro Social, Mexico City, Mexico; 3 Unidad de Investigación Médica en Bioquímica, Centro Médico Nacional Siglo XXI, Instituto Mexicano del Seguro Social, Mexico City, Mexico; 4 Health Care Department, Universidad Autónoma Metropolitana–Xochimilco, Mexico City, Mexico; 5 School of Medicine, Universidad La Salle, Mexico City, Mexico

**Keywords:** long noncoding, metalloproteases, RNA, Takayasu arteritis, vasculitis

## Abstract

**Objectives:**

Takayasu arteritis (TAK) is a large-vessel vasculitis characterized by chronic vascular inflammation and extracellular matrix (ECM) remodeling. Long non-coding RNAs (lncRNAs) have emerged as epigenetic regulators of inflammatory and structural vascular processes. This study aimed to evaluate whether lncRNA HOTAIR (HOX transcript antisense RNA) expression in peripheral blood mononuclear cells (PBMCs) is associated with circulating mediators involved in ECM turnover in patients with TAK.

**Methods:**

Fifty-three patients with angiographically confirmed TAK and 53 age- and sex-matched healthy controls were recruited. HOTAIR expression in PBMCs was quantified by reverse transcription-quantitative polymerase chain reaction. Serum concentrations of matrix metalloproteinase-1 (MMP-1), MMP-2, MMP-3, MMP-9, MMP-13, tissue inhibitor of metalloproteinases-1 (TIMP-1), TIMP-3, extracellular matrix metalloproteinase inducer (EMMPRIN)/CD147, and galectin-3 were measured using enzyme-linked immunosorbent assays.

**Results:**

Compared with controls, TAK patients exhibited significantly higher serum levels of MMP-2 (2245 [1929–2646] vs. 1868 [1576–2355] pg/mL; p=0.036), TIMP-1 (2811 [2644–3216] vs. 2588 [2309–2770] pg/mL; p=0.006), and EMMPRIN/CD147 (1494 [1264–2115] vs. 1310 [1132–1655] pg/mL; p=0.035), along with a reduced MMP-9/TIMP-1 ratio (0.88 [0.61–1.28] vs. 1.18 [0.91–1.41]; p=0.006). HOTAIR expression was markedly downregulated in TAK patients (8.05 [2.52–24.54] vs. 36.09 [30.08–43.85] arbitrary units; p=0.006) and was not associated with disease activity. Notably, HOTAIR expression inversely correlated with circulating MMP-2 (Spearman’s rho -0.276) and EMMPRIN/CD147 (Spearman’s rho -0.280) levels.

**Conclusion:**

TAK displays a circulating proteolytic profile consistent with enhanced ECM turnover and vascular remodeling. The marked downregulation of HOTAIR supports the involvement of epigenetic mechanisms in TAK and suggests a potential association between reduced HOTAIR expression and MMP-mediated vascular injury.

## 1 Introduction

Takayasu arteritis (TAK) is a rare, idiopathic large-vessel vasculitis that primarily involves the aorta and its major branches, typically affecting young individuals. The disease often begins insidiously with nonspecific systemic manifestations and later progresses to vascular insufficiency–manifested as limb claudication, blood pressure asymmetry, and vascular bruits–resulting from progressive arterial stenosis (1). TAK exhibits a marked geographic and ethnic distribution, with the highest incidence reported in Asian and Latin American populations (1). Despite advances in immunosuppressive therapy, many patients progress to irreversible arterial injury (2, 3). Monitoring disease activity remains challenging, as conventional biomarkers such as erythrocyte sedimentation rate (ESR), C-reactive protein (CRP), and clinical indices lack sensitivity for detecting silent vascular inflammation (2, 4). These limitations highlight the need for novel biomarkers that better reflect the underlying vascular remodeling in TAK.

Histologically, TAK lesions demonstrate granulomatous inflammation affecting all arterial layers, accompanied by adventitial fibrosis, elastic lamina disruption, and vascular smooth muscle cell (VSMC) proliferation. These processes contribute to arterial wall thickening, stenosis, and aneurysm formation (5). Proteolytic enzymes, particularly matrix metalloproteinases (MMPs), have been implicated in these structural alterations. Early studies reported increased circulating MMPs in TAK, consistent with degradation of collagen, elastin, and proteoglycans, which are key features of advanced vascular lesions (6). Both MMPs (e.g., MMP-2, MMP-9) and their endogenous inhibitors (tissue inhibitor of MMPs [TIMPs]) orchestrate extracellular matrix (ECM) turnover, and disruption of the MMP/TIMP balance is thought to promote fibrosis and vascular injury in TAK (2, 7).

MMP expression is tightly regulated at transcriptional, post-transcriptional, and epigenetic levels. Long noncoding RNAs (lncRNAs)–transcripts exceeding 200 nucleotides without protein-coding potential–have emerged as significant regulators of cytokine signaling, transcriptional networks, renin-angiotensin-aldosterone signaling, and noncoding RNA cross-talk (8, 9). A recent study revealed dysregulated lncRNAs expression in peripheral blood mononuclear cells (PBMCs) from TAK patients, including downregulation of HOTAIR (HOX transcript antisense RNA) compared with healthy individuals and patients with rheumatoid arthritis (10). HOTAIR is a well-characterized lncRNA that scaffolds the Polycomb Repressive Complex 2 (PRC2) and the LSD1 complex, enabling gene silencing through histone H3K27 methylation and H3K4 demethylation (11). Mechanistically, HOTAIR influences tissue plasticity and ECM remodeling. HOTAIR expression is regulated by transforming growth factor-beta-1 (TGF-β1) via HNF4-α and Smad2/3 signaling, promoting profibrotic protein synthesis (12). Moreover, HOTAIR influences MMP activity through multiple mechanisms, including post-transcriptional regulation of MMP-2 via miR-17-5p sponging and modulation of MMP-13 expression associated with collagen degradation (13, 14). In rheumatoid arthritis, HOTAIR regulates fibroblast-like synoviocytes through the miR-106b-5p/Smad7 axis, thereby influencing MMP-2 and MMP-13 production (15). Collectively, these findings suggest that HOTAIR exerts anti-proteolytic and antifibrotic effects.

Given the central role of ECM remodeling in TAK and the emerging importance of lncRNAs in MMP regulation, we hypothesized that reduced HOTAIR expression may be associated with abnormal protease profiles in TAK. HOTAIR was selected for investigation based on previous observations suggesting altered expression in PBMCs from TAK patients and on extensive experimental evidence linking HOTAIR to epigenetic regulation of genes involved in extracellular matrix remodeling and matrix metalloproteinase activity in several pathological contexts (10, 13–15). Therefore, this study investigated whether HOTAIR downregulation in PBMCs from TAK patients is associated with dysregulated MMP/TIMP expression, thereby exploring a potential epigenetic axis involved in vascular remodeling.

## 2 Patients and methods

### 2.1 Study design

We conducted an observational, cross-sectional study that enrolled consecutive patients with TAK fulfilling the 2022 American College of Rheumatology (ACR)/European Alliance of Associations for Rheumatology (EULAR) classification criteria (16). Given the rarity of TAK and the limited number of eligible patients at a single center, a formal *a priori* sample size calculation was not performed. Instead, we adopted a consecutive sampling strategy and included all ambulatory patients who met the predefined eligibility criteria and were evaluated at our center between January 2023 and September 2023. Eligible participants were required to meet the 2022 ACR/EULAR classification criteria for TAK, present angiographic findings consistent with the diagnosis, and provide written informed consent. Age- and sex-matched healthy individuals without a history of autoimmune or inflammatory diseases were recruited as controls.

During the study period, 53 patients with angiographically confirmed TAK and 53 matched healthy controls were included in the final analysis. Exclusion criteria included active infection, pregnancy, malignancy, and the presence of other autoimmune or systemic inflammatory disorders. At enrollment, TAK patients underwent a comprehensive clinical assessment including medical history, physical examination, laboratory testing, and review of imaging studies. Arterial involvement was classified according to angiographic patterns using the Numano classification. Disease activity was assessed using the Indian Takayasu Clinical Activity Score (ITAS2010) (4).

### 2.2 Ethics statement

The study protocol was approved by the Research and Ethics Committees of the Instituto Nacional de Cardiología Ignacio Chávez (approval number 23-1363) and conducted in accordance with the Declaration of Helsinki. All participants provided written informed consent before inclusion.

### 2.3 Laboratory procedures

Peripheral fasting blood samples were collected in ethylenediaminetetraacetic acid (EDTA)-containing tubes. PBMCs were isolated by Ficoll density-gradient centrifugation (Sigma-Aldrich, St. Louis, MO, USA) and stored at −70 °C in TriPure reagent (Roche, Indianapolis, IN, USA) until RNA extraction. Total RNA was extracted using TriPure reagent according to the manufacturer’s instructions. RNA integrity was evaluated by agarose gel electrophoresis and spectrophotometric purity criteria using a NanoDrop 2000 spectrophotometer (Thermo Fisher Scientific, Waltham, MA, USA). Only RNA samples with an A260/A280 ratio between 1.8 and 2.1 were included in downstream analyses.

Complementary DNA (cDNA) was synthesized from 500 ng of total RNA using the QuantiTect Reverse Transcription Kit (Cat. 205413; Qiagen, Hilden, Germany), which incorporates a genomic DNA elimination step. Briefly, 500 ng of RNA (2 µL) were mixed with 2 µL of gDNA Removal Mix and 11 µL of RNase-free water (final volume 15 µL) and incubated for 2 min at 45 °C. Subsequently, 1 µL of reverse transcription enzyme and 4 µL of reverse transcription mix were added to obtain a final reaction volume of 20 µL. Reverse transcription was performed under the following conditions: 3 min at 25 °C, 10 min at 45 °C, and 5 min at 85 °C.

Quantitative real-time polymerase chain reaction (RT-qPCR) was performed using the QuantiNova SYBR Green PCR Kit (Cat. 208056; Qiagen) on a CFX96 Touch Real-Time PCR Detection System (Bio-Rad, Hercules, CA, USA). Each reaction was carried out in a final volume of 20 µL containing 10 µL of 2× QuantiNova SYBR Green PCR Master Mix, 4 µL of primer assay, 1 µL of cDNA template, and RNase-free water to complete the volume. Primer assays included HOTAIR (GeneGlobe ID: LPH07360A; Qiagen) and GAPDH (GeneGlobe ID: PPH00150F; Qiagen), with GAPDH used as the endogenous reference gene. The amplification protocol consisted of an initial activation step at 95 °C for 2 min followed by 40 cycles of denaturation at 95 °C for 5 s and annealing/extension at 60 °C for 10 s. A melting-curve analysis was performed at the end of the amplification cycles to verify the specificity of the PCR products. Relative gene expression was calculated using the 2ΔΔCt method and expressed as arbitrary units (a.u.) (17). PCR amplification efficiency for each assay was verified using standard dilution curves and was required to fall between 90% and 110% with a correlation coefficient (R²) ≥ 0.99. Samples with Ct standard deviation >0.5 across triplicates were repeated. Relative gene expression levels were calculated using the 2−ΔΔCt method, with normalization to GAPDH as the endogenous control gene.

For protein measurement, blood samples were collected in clot-activator tubes and centrifuged at 600 g for 15 min at 8 °C. Serum was aliquoted and stored at −70 °C until analysis. Levels of MMP-1 (Cat. DY901B), MMP-2 (Cat. DY902), MMP-3 (Cat. DY513), MMP-9 (Cat. DY911), MMP-13 (Cat. DY511), extracellular matrix metalloproteinase inducer (EMMPRIN/CD147; Cat. DY972), TIMP-1 (Cat. DY970), TIMP-3 (Cat. DY973), and galectin-3 (Cat. DY1154) were measured using commercially available enzyme-linked immunosorbent assay kits (R&D Systems, Minneapolis, MN, USA) according to the manufacturer’s instructions. Absorbance was measured using an ELx808 microplate reader (BioTeK Instruments, Winooski, VT, USA). All samples were analyzed in duplicate, and mean values were used for subsequent analyses.

### 2.4 Statistical analysis

Continuous variables are presented as medians with interquartile ranges (IQR; 25th–75th percentile), while categorical variables are expressed as counts and percentages. Comparisons between TAK patients and controls were performed using the Mann-Whitney U test for continuous variables and Fisher’s exact test for categorical variables. For comparisons across three or more ordered groups (e.g., ITAS2010 tertiles), the Kruskal-Wallis test was applied.

To control for multiple testing, p-values were adjusted using the Benjamini-Hochberg false discovery rate (FDR) procedure, with a prespecified FDR threshold of 0.05. Correlations between variables were assessed using Spearman’s rank correlation coefficient with 95% confidence intervals.

A two-tailed p-value <0.05 was considered statistically significant. Statistical analyses were performed using GraphPad Prism v10.6.0 (GraphPad Inc, La Jolla, CA, USA).

## 3 Results

### 3.1 Study participants

The study population comprised 106 individuals, including 53 TAK patients (median age 40 years; 96% female) and 53 healthy controls (median age 42 years; 96% female). Among TAK patients, the median disease duration was 14 (5-23) years. Hypertension was more frequent in TAK patients compared with controls (56% vs. 3%; p <0.001) (**Table 1**).

**Table 1:** Clinical characteristics of study participants.

Characteristic	Takayasu arteritis (n = 53)	Healthy controls (n = 53)	*p*-value
Female – no. (%)	51 (96)	51 (96)	>0.999
Age (years)	40 (34–51)	42 (33–54)	0.790
Disease duration (years)	14 (5–23)	–	
Age at diagnosis (years)	24 (17–32)	–	
BMI (kg/m^2^)	25.1 (21.5–28.3)	26.1 (23.2–28.3)	0.172
Hypertension – no. (%)	30 (56)	2 (3)	**<0.001**
Diabetes – no. (%)	7 (13)	2 (3)	0.160
Smoking – no. (%)	3 (5)	13 (24)	**0.012**

Data are presented as median (interquartile range) unless otherwise specified. Significant *p*-values are in bold.

Definitions: BMI, body mass index.

Stratification of TAK patients according to ITAS2010 tertiles (**Table 2**) revealed that individuals with higher disease activity had greater cardiovascular involvement and more disease flares in the preceding year. No significant differences across tertiles were observed for other clinical manifestations, laboratory parameters, or angiographic subtypes of vascular involvement.

**Table 2:** Disease and laboratory features of patients with Takayasu arteritis, stratified by disease severity.

Characteristic	Disease activity by the ITAS2010 score			*p*-value
	1^st^ tertile (n = 18)	2^nd^ tertile (n = 17)	3^rd^ tertile (n = 18)	
Disease duration (years)	7 (4–22)	16 (6–23)	15 (13–29)	0.145
ITAS2010 score	1 (0–2)	4 (3–5)	7 (6–9)	**<0.001**
Dabague-Reyes score	1 (0–1.5)	1.5 (1.0–2.5)	4.0 (1.1–5.8)	**0.001**
Vascular surgery – no. (%)	7 (38)	6 (35)	6 (33)	0.728
Major organ involvement – no. (%)				
Neurologic	3 (16)	1 (5)	5 (27)	0.374
Ocular	2 (11)	1 (5)	6 (33)	0.075
Cardiovascular	9 (50)	10 (58)	15 (83)	**0.037**
Renal	3 (16)	3 (17)	3 (16)	>0.999
Vascular involvement by Numano – no. (%)				
Type I	1 (5)	0	1 (5)	>0.999
Type II a	1 (5)	3 (17)	0	0.528
Type II b	0	1 (5)	0	>0.999
Type III	0	0	0	>0.999
Type IV	0	0	1 (5)	0.220
Type V	16 (88)	13 (76)	16 (88)	>0.999
Laboratory values				
Hemoglobin (g/dL)	14 (12–15)	13 (13–15)	13 (12–14)	0.683
WBC (1×10^3^ per mm^3^)	7.6 (5.7–9.6)	6.0 (5.4–7.0)	6.6 (5.9–10.3)	0.085
Platelets (1×10^3^ per mm^3^)	278 (230–325)	280 (226–315)	238 (209–325)	0.723
Creatinine (mg/dL)	0.7 (0.6–0.9)	0.7 (0.6–0.8)	0.7 (0.7–0.8)	0.916
Albumin (g/dL)	4.2 (4.1–4.5)	4.4 (4.2–4.6)	4.3 (4.1–4.5)	0.539
hsCRP (mg/L)	1.8 (1.2–4.2)	3.3 (1.2–6.4)	2.9 (1.2–4.2)	0.883
ESR (mm/h)	13 (5–25)	9 (5–18)	15 (11–24)	0.111
SII index	583 (460–1553)	494 (354–700)	760 (473–1069)	0.328
Current drug therapies – no. (%)				
Methotrexate	16 (88)	12 (70)	11 (61)	0.058
Azathioprine	6 (33)	6 (35)	5 (27)	0.721
Cyclophosphamide	0	0	2 (11)	0.080
Prednisone	9 (50)	8 (47)	13 (72)	0.178
Biologics	2 (11)	2 (11)	0	0.207
Flare in the last year – no. (%)	1 (5)	0	6 (33)	**0.013**

Data are presented as median (interquartile range) unless otherwise specified. Significant *p*-values are in bold.

Definitions: ESR, erythrocyte sedimentation rate; hsCRP, high sensitivity C-reactive protein; ITAS2010, Indian Takayasu Clinical Activity Score; SII, Systemic Immune-Inflammation Index; WBC, white blood cells.

### 3.2 Serum extracellular matrix-related mediators

TAK patients exhibited a distinct proteolytic profile consistent with enhanced ECM turnover. Serum levels of MMP-2 (2245 [1929-2646] vs. 1868 [1576-2355] pg/mL; p = 0.009), TIMP-1 (2811 [2644-3216] vs. 2588 [2309-2770] pg/mL; p <0.001), EMMPRIN/CD147 (1494 [1264-2115] vs. 1310 [1132-1655] pg/mL; p = 0.007), and galectin-3 (1257 [906-1821] vs. 1017 [457-1607] pg/mL; p = 0.038) were significantly elevated in TAK patients than in controls (**Table 3**). Conversely, both the MMP-3/TIMP-1 ratio (0.51 [0.40-0.68] vs. 0.55 [0.44-0.76]; p = 0.047) and the MMP-9/TIMP-1 ratio (0.88 [0.61-1.28] vs. 1.18 [0.91-1.41]; p <0.001) were significantly decreased in TAK patients.

**Table 3:** Circulating extracellular matrix-remodeling molecules and HOTAIR expression.

Biomarker	Takayasu arteritis (n = 53)	Healthy controls (n = 53)	Raw *p*-value	Adjusted *p*-value*
MMP-1, pg/mL	2487 (1694-2927)	1941 (1524-2341)	0.056	0.140
MMP-2, pg/mL	2245 (1929-2646)	1868 (1576-2355)	**0.009**	**0.036**
MMP-3, pg/mL	1587 (1173-1986)	1421 (1090-2107)	0.894	0.941
MMP-9, pg/mL	2545 (2109-3519)	2952 (2428-3461)	0.366	0.566
MMP-13, pg/mL	1674 (1302-2252)	1633 (1261-2199)	0.791	0.930
TIMP-1, pg/mL	2811 (2644-3216)	2588 (2309-2770)	**<0.001**	**0.006**
TIMP-3, pg/mL	1314 (948-1994)	1332 (708-2377)	0.427	0.610
EMMPRIN/CD147, pg/mL	1494 (1264-2115)	1310 (1132-1655)	**0.007**	**0.035**
Galectin-3, pg/mL	1257 (906-1821)	1017 (457-1607)	**0.038**	0.126
MMP-1/TIMP-1, ratio	0.81 (0.61-1.07)	0.73 (0.58-0.90)	0.864	0.941
MMP-1/TIMP-3, ratio	1.73 (1.04-2.69)	1.51 (0.71-2.81)	0.598	0.797
MMP-2/TIMP-1, ratio	0.78 (0.66-0.92)	0.75 (0.61-0.94)	0.947	0.947
MMP-2/TIMP-3, ratio	1.68 (1.13-2.68)	1.36 (0.87-2.34)	0.368	0.566
MMP-3/TIMP-1, ratio	0.51 (0.40-0.68)	0.55 (0.44-0.76)	**0.047**	0.134
MMP-3/TIMP-3, ratio	1.36 (0.81-1.67)	1.06 (0.83-1.86)	0.200	0.363
MMP-9/TIMP-1, ratio	0.88 (0.61-1.28)	1.18 (0.91-1.41)	**<0.001**	**0.006**
MMP-9/TIMP-3, ratio	1.95 (1.24-2.86)	2.10 (1.40-3.23)	0.107	0.237
MMP-13/TIMP-1, ratio	0.56 (0.46-0.74)	0.63 (0.52-0.88)	0.666	0.832
MMP-13/TIMP-3, ratio	1.37 (0.84-1.98)	1.31 (0.82-2.05)	0.120	0.240
HOTAIR expression, a.u.	8.0 (2.5-24.5)	36.0 (30.0-43.8)	**<0.001**	**0.006**

Data are presented as median (interquartile range). Significant *p*-values are in bold.

* Benjamini-Hochberg-adjusted *p*-values.

Definitions: EMMPRIN, extracellular matrix metalloproteinase inducer; ITAS2010, Indian Takayasu Clinical Activity Score; MMP, matrix metalloproteinase; TIMP, tissue inhibitor of metalloproteinases.

### 3.3 HOTAIR expression analysis

**Figure 1:**
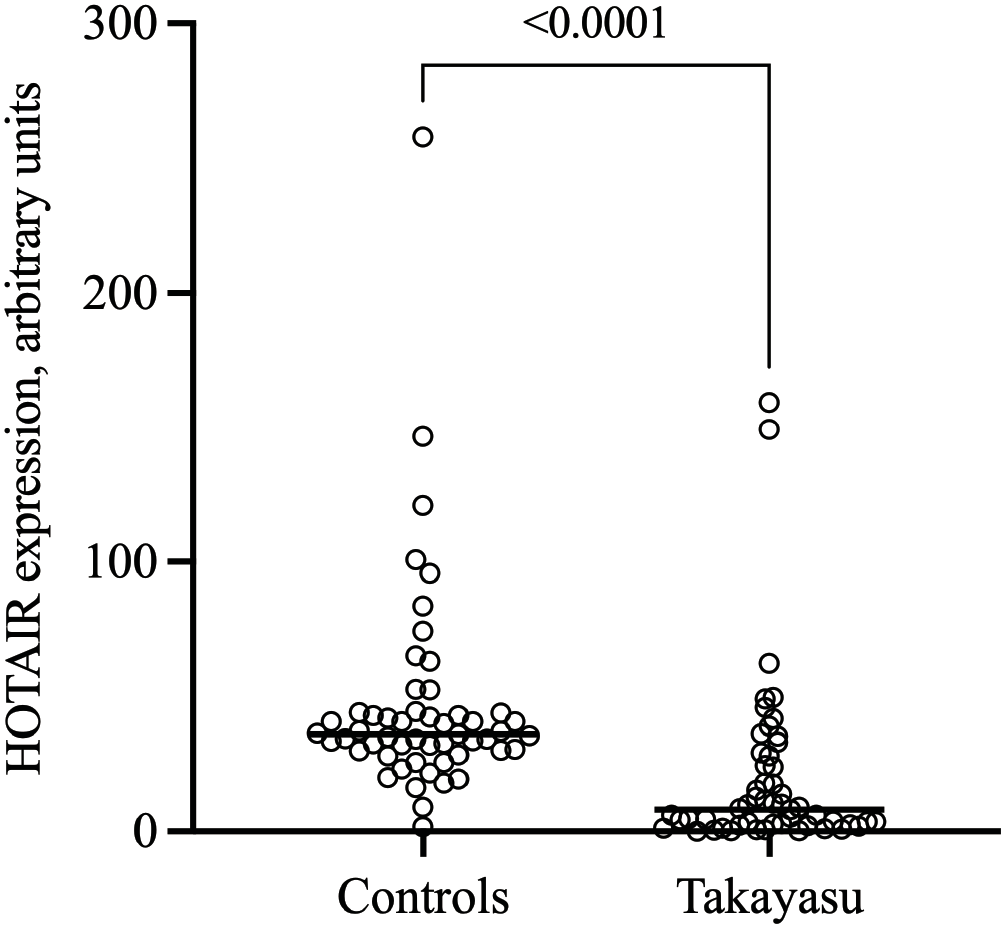
Relative expression levels of the lncRNA HOTAIR in patients with Takayasu arteritis and healthy controls. Scatter plot showing individual values with median value (horizontal line).

HOTAIR transcript levels in PBMCs were markedly reduced in TAK patients compared with controls (8.05 [2.52-24.54] vs. 36.09 [30.08-43.85] a.u.; p <0.001. **Figure 1** and **Table 3**). However, HOTAIR expression did not differ across ITAS2010 tertiles (5.67 [2.64-12.51] vs. 8.54 [3.52-28.09] vs. 9.07 [1.54-29.30]; p = 0.720).

FDR-adjusted analyses identified a restricted set of significant differences between groups. Specifically, TIMP-1, MMP-2, EMMPRIN/CD147, the MMP-9/TIMP-1 ratio, and HOTAIR expression remained significantly different between TAK patients and controls after FDR correction. No other MMPs, TIMPs, or derived ratios retained statistical significance following adjustment (**Table 3**).

### 3.4 Correlation between HOTAIR expression and ECM-related mediators

Correlation analyses performed within the TAK cohort demonstrated moderate-to-low associations between HOTAIR expression and selected ECM-related mediators. HOTAIR levels were negatively correlated with serum levels of MMP-2 (Spearman’s rho –0.276; 95% CI –0.514 to –0.002; p = 0.045) and EMMPRIN/CD147 (Spearman’s rho –0.280; 95% CI –0.518 to –0.002; p = 0.042).

No significant correlations were observed between HOTAIR expression and other ECM-related molecules or measures of disease activity (**Supplementary Table**).

## 4 Discussion

This study demonstrates that TAK patients exhibit significantly reduced HOTAIR expression in PBMCs together with elevated circulating levels of MMP-2, TIMP-1, and EMMPRIN/CD147, suggesting that impaired HOTAIR activity may contribute to dysregulated ECM remodeling in TAK.

A key novel contribution of our study is the observed association between HOTAIR expression and circulating ECM-related molecules. HOTAIR functions as a scaffold for PRC2 and the LSD1/CoREST complex, directing H3K27 trimethylation and H3K4 demethylation to silence target genes (11). Through this mechanism, HOTAIR modulates cell differentiation, inflammation, and ECM homeostasis. Dysregulation of HOTAIR has been widely reported in multiple human diseases, where it modulates the expression of genes involved in ECM dynamics and MMP activity. For example, genetic variants and altered expression of HOTAIR have been associated with susceptibility to several malignancies, including bladder cancer, where HOTAIR dysregulation has been linked to tissue remodeling processes (18). Similarly, in rheumatoid synovial fibroblasts, forced HOTAIR expression suppresses the production of MMP-2 and MMP-13, whereas HOTAIR silencing leads to marked upregulation of MMP-3, MMP-13, and MMP-14 (19). These observations support the concept that HOTAIR participates in regulatory networks controlling ECM turnover across diverse pathological conditions.

In the present study, we observed reduced HOTAIR expression in PBMCs together with increased circulating levels of MMP-2 and EMMPRIN/CD147. However, the mechanistic relationship between these findings remains speculative. Most MMPs detected in TAK lesions are produced locally by infiltrating macrophages and resident stromal cells, including VSMCs and fibroblasts. Consequently, reduced HOTAIR expression in circulating immune cells should not be interpreted as the direct source of circulating MMPs. Rather, altered lncRNA expression in PBMCs may reflect systemic immune dysregulation that indirectly influences vascular remodeling. For instance, dysregulated immune cells may promote macrophage recruitment to the vascular wall, amplify inflammatory signaling pathways, or alter the cytokine milieu within the vascular microenvironment, thereby favoring protease production by local vascular cells. In addition, emerging evidence suggests that regulatory RNAs, including lncRNAs, can be transferred between cells through extracellular vesicles, thereby influencing transcriptional programs in recipient cells (20). For example, exosome-mediated transfer of regulatory RNAs has been shown to modulate inflammatory signaling and matrix remodeling in fibroblasts and endothelial cells (21). Such mechanisms raise the possibility that lncRNAs expressed in circulating immune cells could indirectly influence vascular cell behavior through horizontal RNA transfer (22), although it remains unclear whether these observations represent a biologically meaningful regulatory axis or simply parallel consequences of chronic inflammation.

TAK patients exhibited higher serum levels of MMP-2 and TIMP-1, along with increased concentrations of EMMPRIN/CD147, compared with healthy controls. Elevated MMP-2 is particularly relevant because it degrades basement membrane components and can facilitate aneurysm formation. Increased TIMP-1 may reflect a compensatory response to heightened proteolytic activity. Higher galectin-3 levels are consistent with its recognized profibrotic role, as galectin-3 promotes collagen I deposition and reduces MMP activity via the TGF-β1/STAT3 pathway (23). These findings align with prior reports showing enhanced MMP activity in TAK lesions and extend recent observations on the miR-199a-5p/MMP-2 axis as an inhibitor of the migration and proliferation of VSMCs (6), prompting the intimal infiltration of inflammatory cells into the vascular wall (24). Beyond MMP-2, additional MMPs have also been implicated in TAK. Several groups have reported elevations in MMP-3 and MMP-9, which correlate with both inflammatory activity and structural vascular damage. Matsuyama et al. found that MMP-3 and MMP-9 associate more closely with clinical disease activity, whereas MMP-2 exhibits greater diagnostic accuracy (7). Importantly, MMPs may remain elevated even during clinically “inactive” phases, suggesting that they reflect ongoing subclinical vascular remodeling rather than systemic inflammation. This dissociation from conventional clinimetric indices reinforces the potential value of MMPs as direct markers of tissue injury (2, 4). In our study, the lack of association between HOTAIR expression and the ITAS2010 activity score may therefore indicate that HOTAIR primarily reflects vascular remodeling processes rather than inflammatory activity captured by clinical indices. Integration with vascular imaging modalities, such as PET-CT or angiographic MRI, would help clarify this relationship in future studies.

EMMPRIN/CD147 itself is known to induce MMP synthesis in vascular cells and fibroblasts, amplifying ECM degradation (25). Its elevated levels in TAK, combined with reduced HOTAIR expression, suggest a potential epigenetic mechanism promoting MMP activation. Beyond its role in proteolysis, EMMPRIN/CD147 also promotes monocyte activation and endothelial-lymphocyte interactions, positioning it as a key node between inflammation and structural vascular damage. Evidence from other vasculitides indicate that EMMPRIN/CD147 overexpression drives lymphocytic infiltration and progression to aneurysmal lesions, underscoring its potential contribution in TAK pathogenesis (25).

Mechanistically, reduced HOTAIR expression in TAK could derepress MMP-related genes in both immune and vascular cells. Chronic inflammation itself may downregulate HOTAIR, as proinflammatory cytokines such as tumor necrosis factor and interleukin-1 are known to suppress HOTAIR expression in fibroblasts (19). Thus, HOTAIR reduction likely alters the balance of PRC2-mediated repression, allowing key fibrotic and proteolytic genes to remain epigenetically active. Interestingly, HOTAIR levels in our cohort did not correlate with clinical activity scores, consistent with prior evidence that lncRNAs in TAK do not mirror short-term changes in disease activity (10). One possible explanation is that lncRNAs may require extended periods to exert epigenetic regulation over inflammatory and fibrotic cellular programs, and thus may not be temporally aligned with short-term fluctuations in clinical disease activity (26). Alternatively, the poor performance of current clinimetric indices–particularly when compared with imaging modalities such as positron emission tomography-computed tomography (PET-CT) or magnetic resonance imaging–may contribute to this apparent dissociation. Nonetheless, these imaging techniques have important limitations, including cost, limited accessibility, and, in the case of PET-CT-based approaches, exposure to ionized radiation (2, 4).

Several limitations of this study should be acknowledged. First, the cross-sectional design precludes causal inferences regarding the relationship between HOTAIR expression and ECM remodeling mediators, thereby our findings should be considered hypothesis-generating. Longitudinal studies are required to determine whether changes in HOTAIR expression precede, accompany, or result from proteolytic vascular injury over time. Second, HOTAIR and MMP levels were assessed in peripheral blood rather than directly in vascular tissue, limiting direct extrapolation to processes occurring within the arterial wall. Third, disease activity was evaluated using clinical indices rather than imaging-based modalities, which may be more sensitive for detecting subclinical vascular inflammation and remodeling. Finally, the study focused on a single lncRNA candidate, and broader profiling of noncoding RNAs–including additional lncRNAs and microRNAs–may reveal more comprehensive regulatory networks involved in vascular remodeling in TAK.

Despite these limitations, several strengths should be highlighted. The inclusion of a well-characterized cohort of patients with angiographically confirmed TAK enhances the internal validity and representativeness of the findings. The use of age- and sex-matched healthy controls allowed for robust comparative analyses. Importantly, the simultaneous assessment of HOTAIR expression and a comprehensive panel of ECM-related mediators provided an integrated view of proteolytic imbalance and epigenetic regulation in TAK. The application of FDR correction further strengthens the reliability of the observed associations. Finally, the identification of an inverse relationship between HOTAIR expression and key proteolytic mediators supports a biologically plausible and novel epigenetic-protease axis in TAK, reinforcing the hypothesis-generating value of this work and laying the groundwork for future mechanistic studies, including gain- and loss-of-function experiments in PBMCs or monocyte-derived macrophages to evaluate downstream effects on MMP-2 and EMMPRIN/CD147 expression at the transcript and protein levels.

In conclusion, TAK patients exhibit a circulating proteolytic profile consistent with active ECM degradation. HOTAIR expression is reduced in PBMCs, and lower HOTAIR levels coincide with higher circulating concentrations of MMP-2, EMMPRIN/CD147, and TIMP-1. Together, these findings suggest a previously unrecognized epigenetic mechanism through which HOTAIR may influence MMP-driven vascular remodeling in TAK.

## Data Availability

The raw data supporting the conclusions of this article will be made available by the authors, without undue reservation.
