# Supplementary material for: Altered extracellular matrix remodeling accompanies decreased lncRNA HOTAIR expression in Takayasu arteritis
Source: Front Immunol. 2026 Apr 16;17:1800351. doi: 10.3389/fimmu.2026.1800351 (PMC13128639; doi:10.3389/fimmu.2026.1800351)
Supplement: Supplementary file 1 [file Table1.docx]

**Supplementary Table. Correlation of HOTAIR expression with extracellular matrix remodeling markers and disease activity in Takayasu arteritis**

|  | Spearman’s ρ coefficient | 95% confidence interval | *p*-value |
| --- | --- | --- | --- |
| MMP-1 | -0.101 | -0.368 to 0.182 | 0.471 |
| MMP-2 | -0.276 | -0.514 to -0.002 | **0.045** |
| MMP-3 | -0.084 | -0.354 to 0.198 | 0.546 |
| MMP-9 | -0.081 | -0.351 to 0.201 | 0.564 |
| MMP-13 | 0.127 | -0.156 to 0.391 | 0.364 |
| TIMP-1 | 0.002 | -0.276 to 0.280 | 0.987 |
| TIMP-3 | 0.198 | -0.084 to 0.451 | 0.154 |
| EMMPRIN/CD147 | -0.280 | -0.518 to -0.002 | **0.042** |
| Galectin-3 | 0.013 | -0.265 to 0.290 | 0.923 |
| ITAS2010 score | -0.004 | -0.282 to 0.274 | 0.977 |

Significant *p*-values are in bold.

Definitions: EMMPRIN, extracellular matrix metalloproteinase inducer; ITAS2010, Indian Takayasu Clinical Activity Score; MMP, matrix metalloproteinase; TIMP, tissue inhibitor of metalloproteinases.
